# FMR1 Premutation Is an Uncommon Explanation for Premature Ovarian Failure in Han Chinese

**DOI:** 10.1371/journal.pone.0103316

**Published:** 2014-07-22

**Authors:** Ting Guo, Yingying Qin, Xue Jiao, Guangyu Li, Joe Leigh Simpson, Zi-Jiang Chen

**Affiliations:** 1 Center for Reproductive Medicine, Shandong Provincial Hospital Affiliated to Shandong University, Shandong University, National Research Center for Assisted Reproductive Technology and Reproductive Genetics, The Key laboratory for Reproductive Endocrinology of Ministry of Education, Shandong Provincial Key Laboratory of Reproductive Medicine, Jinan, China; 2 Research and Global Programs March of Dimes Foundation, White Plains, New York, United States of America; 3 Human & Molecular Genetics, Obstetrics and Gynecology, Herbert Wertheim College of Medicine, Florida International University, Miami, Florida, United States of America; 4 Renji Hospital, Shanghai Jiao Tong University School of Medicine, Shanghai, China; University of Science and Technology of China, China

## Abstract

**Background:**

In premature ovarian failure (POF), cessation of menstruation occurs before the expected age of menopause. Approximately 1% of women are affected. FMR1 premutation was reported to be responsible for up to 3.3%–6.7% of sporadic POF and 13% of familial cases in Caucasians, while the data was absent in Chinese population. Therefore, the impact of FMR1 CGG repeat on ovarian reserve is needed to be investigated in large Chinese cohort.

**Methods:**

The number of FMR1 CGG repeat was determined in 379 Han Chinese women with well-defined 46, XX non-syndromic sporadic POF and 402 controls. The age of menopause onset in respect to CGG repeats was further analyzed.

**Results:**

The frequency of FMR1 premutation in Han Chinese POF was only 0.5% (2/379), although it was higher than that in matched controls (0%, 0/402), it was much lower than that reported in Caucasian with POF (3.3%–6.7%). The prevalence of intermediate FMR1 (41–54) was not increased significantly in sporadic POF than that in controls (2.9% vs. 1.7%, P = 0.343). However, POF patients more often carried a single additional CGG repeat in a single allele than did fertile women (allele-1: 29.7 vs. 28.8, P<0.001; allele-2: 32.6 vs. 31.5, P<0.001). POF patients with both alleles of CGG repeats outside (below or above) the normal range (26–34) showed an earlier age of cessation of menses than those with two alleles within normal range (hom-high/high vs. norm: 20.4±4.8 vs. 24.7±6.4, p<0.01; hom-low/high vs. norm: 18.7±1.7 vs. 24.7±6.4, p<0.01).

**Conclusions:**

FMR1 premutation seems to be an uncommon explanation for POF in Han Chinese. However, having both alleles with CGG repeats outside the normal range might still adversely affect ovarian aging.

## Introduction

Fragile X mental retardation 1 (FMR1) is an X-linked gene carrying dynamic triplet CGG repeats in its 5′ untranslated region [1]. Alleles with ≤40 CGG repeats are traditionally considered normal [2]. The full mutation, in which alleles have >200 CGG repeats, causes transcriptional silence of FMR1 gene, absence of the fragile X mental retardation protein (FMRP) and well characterized features of the fragile X syndrome, which was the most common cause of inherited mental retardation and autism [3]. Between the normal (29–31) and full mutation (>200) range, there are intermediate and premutation range, both unstable and capable of expanding to full mutation over several generations [2]. The premutation range starts with 55 repeats and ends at 199 repeats; the intermediate range remains controversial, but generally is considered 41 or 45 to 54 repeats [2], [4].

FMR1 premutation (55–199) is accepted as a relatively common explanation for altered ovarian function and loss of fertility [5]. In mouse model carrying FMR1 premutation, reduced number of growing follicles and impaired female fertility was observed and a reduced phosphorylation of Akt and mTOR proteins was elucidated [6]. In Caucasians, the prevalence of premature ovarian failure (POF) in FMR1 premutation carriers is significantly higher than that in general population (13%–26% vs. 1%) [2]. FMR1 premutation occurs more frequently in POF patients having a positive family history compared to sporadic ones (13%–16% vs. 1.6%–3.0% in British; 12.1% vs.10% in Italian) [7]–[11]. However, in POF patients from Hong Kong (China) and Japan, the frequency of FMR1 premutation was only 0.9% (1/116) and 1.6% (2/128), respectively [4], [12]. Another two studies in Indian, including 80 and 289 POF patients respectively, revealed none of them carrying FMR1 premutation [13], [14]. These data indicated that fewer patients with POF from Asian than Caucasian carried FMR1 premutation. Barasoain et.al found intermediate FMR1 (35–54 CGG repeats) associated with diminished ovarian reserve [15]. Gleicher and colleagues believe that having less than intermediate (41–54) but greater than the accepted normal (29–31) CGG repeats deleteriously affects ovarian ageing [16], [17], perhaps influencing the ovarian response [18] and adversely affecting outcome during IVF-ET treatment [19]. In contrast, Lledo [20], Bennett [21] and Murray [8] found that in Spanish and British populations neither ovarian reserve nor ovarian response was adversely affected by intermediate or normal high sized CGG repeat. These contradictions led us to wonder how the FMR1 mutations in Chinese women with POF, and furthermore the clinical significance of FMR1 tests.

## Materials and Methods

### Study population

In this control cohort study, we investigated 379 sporadic POF patients and 402 matched controls for the number of triple CGG repeats in both alleles of FMR1 gene. Inclusion criteria for POF consisted of cessation of menstrual cycles before 40 years of age, with at least twice serum follicle stimulating hormone (FSH) concentrations exceeding 40 IU/L. Women with known chromosomal abnormalities, previous ovarian surgery, chemotherapy, radiotherapy or familial member diagnosed with POF were excluded. Information was sought on associated somatic anomalies, specifically adult onset neurologic disorder, referred to as fragile X–associated tremor/ataxia syndrome, or mental retardation. The 402 controls were recruited from a cohort for health checkup. They were known to be menstruating regularly, had normal FSH levels and normal pelvic ultrasound imaging. Clinical features of POF patients and controls were shown in Table 1.

**Table 1:** Clinical characteristics of POF patients and controls.

Characteristics	POF	Control	
		<40 yrs	40–44 yrs
**No.**	379	378	24
**Age (yrs)**	31.7±4.2	30.5±4.4	41.1±1.4
**Age at menarche (yrs)**	14.5±1.8	14.9±1.5	15.2±1.5
**Age at onset of menstrual dysfunction (yrs)**	23.3±6.5	-	-
**Age of amenorrhea (yrs)**	25.1±5.6	-	-
**Age at diagnosis (yrs)**	30.1±4.8	-	-
**FSH (IU/L)**	77.0±27.4	7.4±1.8	8.2±2.6

Note: All of the normal control women did not have amenorrhea.

Informed consents were signed by all subjects. This study was approved by the Institutional Review Board of Reproductive Medicine of Shandong University.

### Genotype analysis

With the genomic DNA extracted from peripheral blood lymphocytes, the FMR1 gene was amplified by PCR with fluorescently labeled primers [1], [22], and the CGG repeats of two alleles were counted by capillary electrophoresis (CE) on an ABI 3730 instrument (Applied Biosystems, Madrid, Spain). If only one allele was identified, a rich CGG repeat primed (RP) PCR was performed using AmplideX FMR1 PCR Kit (Asuragen, USA), and the PCR products detected by CE were analyzed using Gene Mapper 4.0 software (Applied Biosystems).

Referring to the nomenclature of Gleicher, et al., the allele with lower number of CGG repeats was defined as allele-1, whereas the one with a higher number was defined as allele-2 [16]. The normal range was defined as 26–34 CGG repeats and the designations of FMR1 genotypes under discussion here referred to the classification rules raised by Gleicher, et al [23]: normal (*norm*)– both alleles within normal range; heterozygous (*het*)– one allele within normal range and the other one outside, further stratified as *het-norm/low* and *het-norm/high*, according to the abnormal allele being below or above normal range; homozygous (*hom*)– both alleles outside normal range, further stratified as *hom-low/low*, *hom-high/high* and *hom-low/high* according to the rules took above (Table 2).

**Table 2:** Distribution of FMR1 genotype and sub-genotype in sporadic POF patients.

		Allele-1	Allele-2	POF (n = 379)
***norm***		26–34	26–34	256 (67.5%)
***het***	*het-norm/low*	<26	26–34	20 (5.3%)
	*het-norm/high*	26–34	>34	90 (23.7%)
	Total			110 (29.0%)
***hom***	*hom-low/low*	<26	<26	1 (0.3%)
	*hom-high/high*	>34	>34	9 (2.4%)
	*hom-low/high*	<26	>34	3 (0.8%)
	Total			13 (3.5%)

Note: By definition allele 1 has the lowest number of repeats. *norm*: both alleles within normal range; *het*: one allele within normal range and the other allele outside; *hom*: both alleles outside normal range.

### Statistical analysis

SPSS 19.0 computer software (IBM, Armonk, NY, USA) was used for data analysis. The continuous data were checked for normality and described as mean ± SD or mean (95% confidence interval for mean). Numerical data were analyzed by independent sample T-tests or Mann-Whitney Test, and Pearson chi-square test or Fisher's exact test was used to test categorical data. All the P values were two-sided, and P<0.05 was considered statistically significant.

## Results

**Figure 1:**
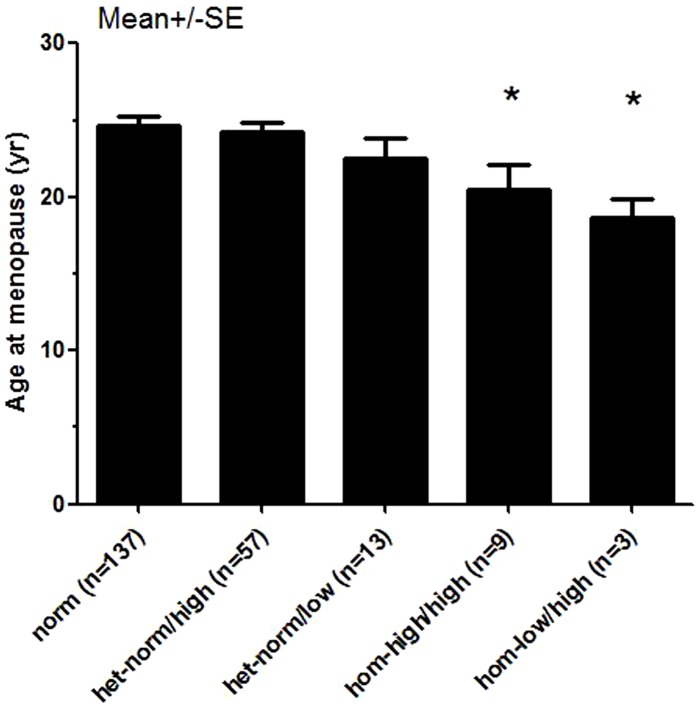
POF patients with sub-genotype *hom-high/high and hom-low/high* showed earlier age at menopause compared with *norm* (20.4±4.8 vs. 24.7±6.4, p<0.01; 18.7±1.7 vs. 24.7±6.4, p<0.01, respectively).

Compared with controls, both allele-1 and allele-2 in POF cases demonstrated a small but significant shift towards a single additional CGG repeat (29.7 vs. 28.8, P<0.001; 32.6 vs. 31.5, P<0.001, respectively) (Table 3). FMR1 premutation (55–199) was observed in only two POF patients (2/379, 0.5%) and none of normal matched controls. The prevalence of intermediate FMR1 (41–54) in POF cases was not increased significantly compared with controls (2.9% vs. 1.7%, P = 0.343) (Table 3). POF patients with sub-genotype *hom-high/high and hom-low/high* showed earlier age at menopause compared with *norm* (20.4±4.8 vs. 24.7±6.4, p<0.01; 18.7±1.7 vs. 24.7±6.4, p<0.01, respectively) (Figure 1).

**Table 3:** Comparation of the CGG repeats on FMR1 gene between sporadic POF patients and normal control women.

	POF (n = 379)	Control (n = 402)	P-value
**Allele-1**	29.7 (29.5–30.0)	28.8 (28.6–29.0)	<0.001^a^
**Allele-2**	32.6 (32.1–33.1)	31.5 (31.1–31.8)	<0.001^a^
**Unaffected(≤40; n%)**	366(96.6)	395(98.3)	0.174^b^
**Intermediate (41–54; n%)**	11(2.9)	7(1.7)	0.343^b^
**Premutation (55–200; n%)**	2(0.5)	0	-
**Full mutation (>200; n%)**	0	0	-

Note: P-value was obtained by Mann-Whitney Test^a^ or Pearson chi-square test^b^ between POF and Control.

## Discussion

Investigating the largest POF cohort of Han Chinese females accumulated to date, we initially determined the frequency of FMR1 premutation to be only 0.5% (2/379), much lower than that previously reported in Caucasian cohorts: British (1.6–3%) [7], [8], [10], Italian (3.3–10%) [9], [11], Polish (7.9%) [24], Turkish (4.9%) [25] and Slovenian samples (4.8%) [26]. The premutation frequency was 1.6% in the only reported Japanese study [4], and 0.9% in the smaller Chinese study [12]. Therefore, FMR1 premutation seems to be an uncommon explanation for POF in Han Chinese. Furthermore, there was no significant difference with respect to intermediate FMR1 between POF cases and controls (2.9% vs. 1.7%, P = 0.343). Similar prevalence has been drawn from Japanese POI cohorts (3.9%) [4], indicating that intermediate FMR1 does not contribute to POF in Chinese either [8], [21]. However, in some Caucasian studies the effect of intermediate FMR1 on ovarian function was controversial. Barasoain and Gleicher et al. found normal high sized and intermediate FMR1 (35–54 CGG repeats) associated with diminished ovarian reserve [15]. In contrast, Lledo [20], Bennett [21] and Murray [8] found that in Spanish and British populations neither ovarian reserve nor ovarian response was adversely affected by intermediate or normal high sized CGG repeat. The contradiction might be attributed to ethnic differences and the bias of recruited participators in each study.

In our study, the distribution of FMR1 genotypes in sporadic POF patients was similar to that previously reported in Asian cohort [27]. POF patients with sub-genotype *hom-high/high and hom-low/high* showed an earlier age of onset of menopause compared with those with *norm* (20.4±4.8 vs. 24.7±6.4, p<0.01; 18.7±1.7 vs. 24.7±6.4, p<0.01, respectively) (Figure 1). That consist with Gleicher's conclusion [19], [28], which suggested that not only abnormally higher CGG repeat numbers have deleterious effect on ovarian aging but also abnormally lower numbers. Our results indicated that having both alleles, rather than a single allele, with CGG repeats outside (above or below) the normal range might adversely affects ovarian function and presumably ovarian reserve in Han Chinese. Considering the relatively small sample size of the sub-genotype *hom-high/high (n = 9) and hom-low/high (n = 3)*, more studies with larger sample size are needed.

The seemingly contradictory findings of a low sporadic FMR1 premutation carrier rate (0.5%) and no increased prevalence of intermediate FMR1 in Han Chinese POF, the deleterious effect of a single additional CGG repeat are not well explained. However in other dynamic mutations (e.g. Huntington disease, myotonic dystrophy, spino-bulbar muscular atrophy), the number of amplified repeats needed to exert a phenotypic effect is not much larger than that considered normal. For example, in Huntington disease, deleterious effects are manifested with more than 36 CAG repeats, whereas 8–35 repeats is normal [29]. In myotonic dystrophy type 1, >50 CTG repeats is abnormal, while 5–37 repeats normal [30]. In spino-bulbar muscular atrophy, >40 CAG repeats is abnormal, but 14–32 repeats normal [31]. In Fragile X syndrome the wide dichotomy between modal number of CGG repeats (29–31) and the >200 required for mental retardation is the exception. By contrast, considering POF cases having CGG repeats outside normal but below intermediate range showed relatively earlier age at menopause, ovarian disturbance may be sensitive to small changes in CGG repeats, more analogous to observations in Huntington disease and myotonic dystrophy type 1.

In summary, FMR1 premutation was not a common explanation for sporadic POF in Han Chinese. But in some familial POF patients, male or female relative with mental retardation could be observed; thus, POF or fragile X tremor/ataxia syndrome could arise in the next generation of familial POF patients. Therefore, from a clinical perspective our results do not obviate the need for FMR1 testing in a woman having POF, even Chinese. Yet, pause may be in order before embarking on general population screening programs in Chinese or Asian populations in the absence of a positive family history.
